# CMLLite: a design philosophy for CML

**DOI:** 10.1186/1758-2946-3-39

**Published:** 2011-10-14

**Authors:** Joe A Townsend, Peter Murray-Rust

**Affiliations:** 1 Unilever Centre for Molecular Science Informatics, Department of Chemistry, Lensfield Road, Cambridge, CB2 1EW, UK

## Abstract

CMLLite is a collection of definitions and processes which provide strong and flexible validation for a document in Chemical Markup Language (CML). It consists of an updated CML schema (schema3), conventions specifying rules in both human and machine-understandable forms and a validator available both online and offline to check conformance. This article explores the rationale behind the changes which have been made to the schema, explains how conventions interact and how they are designed, formulated, implemented and tested, and gives an overview of the validation service.

## Introduction

There is an on-going need for formal, computable representations of scientific data and documents which are also accessible to humans [1-3]. The challenge is to devise systems that people will not only use but for which they will, critically, develop additional tools and content. Our approach for chemistry is Chemical Markup Language (CML) (whose evolution and philosophy is described elsewhere in this issue [4]) which has been developed to support five main areas of chemistry (molecules, reactions, solid-state, spectroscopy and computational chemistry).

The strengths and weaknesses of CML have been recently analysed by Dumontier [3] and we quote directly:

*Chemical Markup Language, backed by a controlled vocabulary, has been rather successful in specifying most aspects of chemistry, from small molecules and their connectivity to polymers and crystal structures*.

*Unfortunately, while most elements of this specification can be parsed out using one of the many XML libraries, certain elements do not render themselves to facile interpretation. Consider the sample CML specification of a water molecule [...]. In order to identify the member atoms in a given bond, it is necessary to carry out string processing as an intermediate step. Further, while many of the elements of CML are defined in a controlled vocabulary, the lack of explicit, consistent, and formal axiomatization of the involved concepts gives rise to difficulties in inferring connections between chemical concepts where no such connections are stated explicitly, something that is possible in formal ontology-backed RDF-based information specifications. Although CML specifications have been increasingly evolving to incorporate elements of the Semantic Web, the lack of widespread adoption of the format, and the limited availability of large-scale CML-based chemical knowledge repositories, have somewhat limited CML-assisted federation of the world of chemical data. Furthermore, the implementation of coverage of additional chemical concepts in most chemical representations requires a formal, rigorous representation specification, complicating the incorporation of data represented using domain-specific representation extensions. We believe that an ideal chemical representation would require no specialized wrapper or interpreter, would be generic such as to allow for facile and conflict-free extensions, would be based on a formal ontology, and would be encoded in a machine-*understandable *(as opposed to simply machine-readable, as in CML) manner and therefore facilitates automated reasoning and data integration*.

This article addresses these points and describes a system we have built for managing explicit and implicit semantics. It was initially developed during the Chem4Word (C4W) project [5] (which creates or edits CML documents in a.NET/Word context) and which we have now generalised to any CML deployment. In C4W we agreed that a fundamental part of the design was that the semantics could be verified. Any document input to the system must be semantically valid so that the C4W system would not break for invalid input. Essentially we designed a contract between the importing system, and the editing/display system.

Rather than rewrite JUMBO [6] and other CML libraries, we designed a set of rules for conformant input documents and tools to process validation. These tools (CML schema3 and CMLValidator) are platform-independent and are reported in this article.

### Semantics in CML

We agree with Dumontier's analysis and in this article show how our current approach to semantics in CML is both achievable and largely compatible with his and others' [7,8] ideal chemical representations. As noted, CML has a small, but important, set of elements (molecule, atom, bond, crystal, spectrum and a few others) where some semantics are implicit and the rules hardcoded. This approach is pragmatic; translating the implicit rules to formal semantics is a considerable effort and makes it more difficult to write libraries to support them.

**Figure 1:**
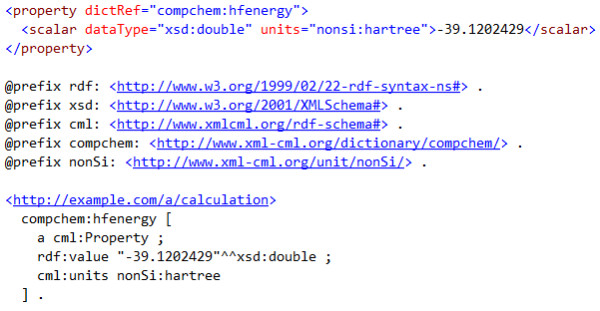
**A property represented in CML (top) and the equivalent RDF (bottom)-as used in the Quixote repository **[47]. The dictionary is referenced by **compchem:hfenergy **for which there must be an entry in an online dictionary. These are completely equivalent and can be translated in both directions without semantic loss. (There are minor syntactic variants such as the capitalization varying systematically.) It is always possible to generate RDF from CML; the reverse may not be possible for arbitrary RDF. The challenge is to create communally acceptable dictionaries/ontologies-the syntax (CML or RDF) is immaterial.

However most CML concepts can be automatically expressed in equivalent semantic form, *e.g*. using RDF format [9] for the document and RDFSchema [10] or (if appropriate) the OWL language to specify an ontology [11] (see Figure 1) and managed with generic (non-chemical) semantic tools. The use of RDF in this manner is advocated by *e.g*. the Bio2RDF project [12]. In particular property and parameter can be completely represented in RDF and we already use this extensively in Quixote [13,14] and similar projects (where CML is imported as RDF).

We have explored a full RDF implementation of CML through ChemAxiom [15] (an OWL-compatible representation of physical chemical properties) and we have also explored full RDF in Open Bibliography [16]). Both of these have shown that the entry overhead is high as the tools are at an early stage. For example there is no support for RDFSchema-based approaches in chemistry. At this stage in chemical informatics, therefore, we feel that CML as a mixture of explicit and implicit semantics provides a useful infrastructure accessible to a large number of implementers and users.

### Implicit semantics

As a typical example of implicit semantics CML schema2.4 requires the formalCharge on a molecule to be consistent with the formalCharges on descendant molecules and atoms. We can express this in pseudocode:

if (not molecule[@formalCharge]) then

molecule@formalCharge: = =

sum (./molecule@formalCharge *./molecule@count) +

sum (./atomArray/atom(@formalCharge * @occupancy * @count))

(If the formal charge (an integer) is missing on a molecule, calculate it by recursively summing its descendants. This is more complex in practice as we have to apply semantics for atoms without formalCharge.)

CML has thousands of relationships like this, and they are relatively straightforward to implement through procedural code (in libraries such as JUMBO, Chem4Word, the Chemistry Development Kit (CDK) [17], Open Babel [18]*etc*.). This article describes how their combination with unit tests and other validation procedures creates strong accessible semantics.

### Choice of semantic system

It is commonly believed that the (perceived) ease of use of a new technology will affect its adoption by communities [19]. A successful deployed system needs to have the following interconnected components:

• Accessibility for humans

• Proven infrastructure

• Authoring tools

• Reading tools

• Editors

• Domain libraries

• Critical mass of content

• Agreed concepts and vocabularies

• Critical mass of users.

This requires a large investment to which we also have to add *Postel's Law *[20]: "be conservative in what you send, liberal in what you accept" *i.e*. do extra implementation work to make it forgiving to use. However we believe the investment in CML [44] has been sufficient to make the semantic approach valuable and tractable.

There seems to be a conservation law which trades ease of implementation and deployment for semantic power. At one of the spectrum is natural language (NL) which is almost infinitely expressive. It relies on an implicit fluid vocabulary and the burden on interpretation is almost completely on the accepter. Its flexibility also generates ambiguity. At the other end are completely hardcoded unambiguous systems with very limited scope (such as InChI [21]); this works because there is a single global implementation of a canonical InChI generator. NL can transmit the concept of "boiling point" because "everybody knows what a boiling point is"; InChI cannot represent the concept at all. To represent "boiling point" formally, however, is by no means trivial-we have to think about units, pressure, error estimations, *etc*. Does boiling point apply to vapour- > liquid transitions? There are thousands of similar chemical concepts all of which must be formalized. There is no escape from this labour.

CML trades full semantic representability for (relative) ease of implementation together with clarity for humans. CML takes a pragmatic view that a large number of chemical concepts are implicitly very well understood (most were formulated 100+ years ago) and the semantics can be hardcoded. This allows us to write software libraries for analysing orbital energies, balancing reactions, finding moments of inertia, *etc*. using the common representation that CML provides.

Here we explore how CML, which represents a set of basic chemical "nouns" (objects), can be combined in flexible, yet rigorous ways. In particular it has to be possible to write software systems that support these developments. We do not set *a priori *constraints on how these nouns can be used, but we require that these usages are documented and validatable, allowing us to write conformant software for each usage.

CML deliberately does not attempt to represent relationships between objects leaving that to RDF; nor does it represent processes (we are still searching for a good, common, formulation). CML is designed to interoperate with other markup languages (XHTML [22], MathML [23], SVG [24], *etc*.) and is incorporated in some approaches, *e.g*. BioPAX [25].

At the present time, therefore, CMLLite represents a cost-effective system which can validate a wide range of chemical documents.

### Community requirements and CMLLite conventions

CML is now largely developed by communities who build prototypes and provide feedback on how well they work; CMLLite has been created and deployed in this way (Table 1).

**Table 1:** Current communities in CML.

Convention	requires	status	Notes
unitType		complete	"standard" for all CML unitTypes

unit	unitType	complete	"standard" for all CML units

Dictionary	unit	complete	"standard" for all CML dictionaries

Molecular	dictionary	beta	"simple" molecules without properties or spectra

Compchem	molecular	alpha	Computational chemistry, especially for Quixote

CMLComp [26]		implicit	solid-state software

CMLSpect [27]		implicit	Spectra consistent with JSpecView [28]

Note: CMLComp and CMLSpect formulated a set of rules but do not have explicit CMLLite validators.

The greater flexibility introduced with CML schema3 allows users to create valid documents almost as they want but requires a greater effort understanding for both humans and machines to understand the document. Here are typical community requirements:

• CMLSpect. "All spectra MUST contain x-data and y-data".

• CMLComp. "only the following CML elements are allowed: module, molecule, atom, property,..." "bond MUST NOT appear as it is not a QM concept"

• molecular (from the Chem4Word project). "a bond MUST contain references to two distinct atoms, the atoms MUST exist, and be in the same ancestor molecule".

• compchem (from Quixote). "a document MUST have a list of jobs, and each job MUST describe environment, initialization, calculation, and finalization". All molecules MUST obey the molecular convention.

• dictionary. "all entries MUST have a definition and MAY have one description."

• Unit-dictionary. "there SHOULD be a specific dictionary for SI units and unitTypes."

The terms are used as in the IETF's RFC 2199 [29]: "MUST", "MUST NOT", "REQUIRED", "SHALL", "SHALL NOT", "SHOULD", "SHOULD NOT", "RECOMMENDED", "MAY", and "OPTIONAL". This approach is central to CMLLite.

These domains of chemistry think about chemistry differently from each other; often this means a very tight specification of rules in one particular area of expertise and very little if any applied to the rest. The loosening of the content model in schema3 allows users to combine the elements and attributes as they need. However, users still need to be able to specify a set of rules (constraints) which model their particular domain. The entire set of constraints which the CML should conform to is called a *convention*. Every convention requiring another recursively inherits (aggregates) the requirements from that convention.

A convention should be the result of community engagement and discussion reflecting historical practice and experience. The social aspects of the process of agreeing conventions are discussed in the companion articles [13,30].

A convention is:

• A description to a human reader of the purpose of the convention, its scope and its implementation. A human MUST be able to hand-craft a compliant document by reading the specification.

• A description to an implementer of exactly how software SHOULD, MAY, MUST and MUST NOT behave when given any possible input. For example software validating a document purporting to be compliant to a particular convention MUST raise an error if it encounters a node defined in the convention but used incorrectly. If it encounters a node not in the convention, its behaviour is undefined but the default should be to inform the user.

• A statement of interest in a particular subset of CML by a community.

The prime purpose of the convention is validation of documents before transferring them to software. As a result the software is more straightforward to implement and test.

### The Need for Validation

**Figure 2:**
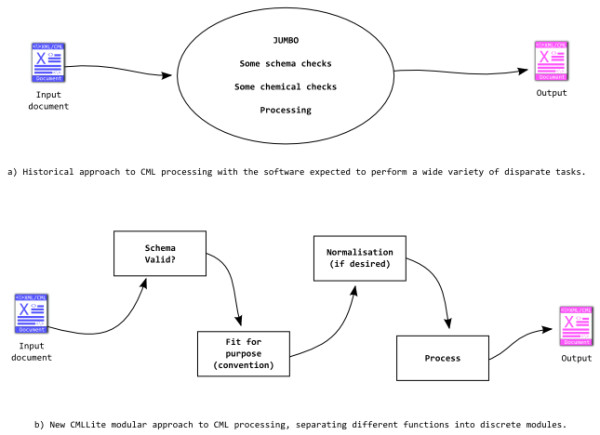
**(a) historical approach to CML processing**. Software was expected to perform a wide variety of tasks including validation and transformations (processing). (b) the CMLLite approach: each module performs only one task *i.e*. validation, normalisation or transformation (processing). This makes each of the modules more straightforward to understand and produces cleaner code.

Validation of input documents is at the heart of the CMLLite approach. There are two complementary approaches to validation (see also Figure 2). Both components (Schema and convention) are *validators *and are normally run sequentially

• **XSD Schema **[31,32]. CML has used this for many years. It works well for isolated elements and attributes with uncomplicated child content. It breaks or is inappropriate for several chemical concepts, complicated content and relationships. In this article we report schema3 where many of the broken and inflexible constraints have been removed. Note that all CML documents now should validate against schema3.

• **Conventions**. These add power to schema3 and allow many complicated concepts to be represented (in XSLT [33]/XPath [34]).

These components are now described in more detail.

### CML Schemas-Evolution to schema3

In themselves, schema constraints can provide little chemical validation but provide good support for other simple concepts (*e.g*. numeric, date, and containers) and are the platform on which further constraints are built.

#### Content Models

Schema2.4 introduced flexibility through more relaxed content models than previous incarnations (an unordered child set with no enforced cardinality), and re-usable attributes. Schema3 has even more flexible content (effectively "any" for most elements) and much of the burden of validation has been devolved to conventions. Specific issues are described below (and are also addressed in the CML retrospective paper). The move away from the 'one-size-fits-all' model imposed by schema2.4 to a more modular, flexible approach, with supporting tools for implementation, has been driven by challenges in the general areas listed below in approximate order of importance:

• **Content Model **(what elements are allowed as children of which elements). Schema3 explicitly removes as much of the content model as possible.

• **Attribute names and attributeGroups**. Schema2.4 allowed attributes to be defined independently of elements. For maintenance purposes each attribute was defined in its own *attributeGroup*. Unfortunately some attributes used polymorphic names (*e.g*. "type") and were not re-locatable. The desire to maintain backward compatibility with the majority of existing software means that we were unable to redesign the attribute names..

• **Union of enumerated values**. Some attributes (and string content) used the XSD union approach to express both controlled (enumerated) and uncontrolled vocabulary. Here enumerated values are of one data type whilst the UNIONed value is of a different type, and has to be processed differently. The CMLLite approach restricts attribute values to a single data type, and uses the dictionary (dictRef) mechanism to provide additional information as required.

• **Mixed content **(text and element children). This was used to support free text but is technically challenging and we are deprecating its use in favour of (say) XHTML constructs.

• **Aliases **(*e.g*. '1' and 'S' for the order of single bonds). These cause a huge overhead in software, are deprecated, and will trigger warnings when the CML is validated against the validation service based on schema3. Normalisation is advised at this stage.

CML has grown to have a collection of approximately 100 elements and approximately 100 attributes. Most of these are in common use, but there are very few documents which use more than about 20 elements and 20 attributes at a time. For example a solid-state calculation has relatively little in common with the textual report of a chemical synthesis. Most elements in CML can be used independently of most other elements and schema3 explicitly supports this. For example a spectrum might occur with a molecule, with a crystal structure or with a computational chemistry output. Some elements have a more restricted use, for example bonds and atoms normally only occur within the context of a molecule. Attributes are more varied, in that some are specific to particular elements (*e.g*. atomRefs2 normally only occurs on the bond elements) while others are very generic (*e.g*. title, id, dictRef). The CML schema determines some of the pattern of attribute occurrence, but leaves others up to the individual conventions.

Almost all changes are backward-compatible as schema3 is more forgiving than schema2.4; a few elements contained mixed content and have been obsoleted (annotation, appinfo, documentation, relatedEntry).

#### Attributes

Attributes define string values and can constrain syntax, dataTypes, lists and other constructs. In schema2.4 many attributes had data types defined by a union of enumerations and a "namespaceRef" pattern (effectively a QName). This has now been relaxed to the enumeration with the addition of "other". Constraints are then added with XPath/XSLT rules. The polymorphism of attributes with names such as type (Appendix A) has not yet been addressed. In schema3 attributes are used in the same way as in schema2.4 and rely on additional constraints added through conventions. Elements with text-only content (scalar, array, matrix) are polymorphic (*e.g*. can be numeric, string, date) and are not supported well by schema constraints.

### Conventions

A convention specifies and can enforce the relationships between schema components and consists of (often a large number of) statements (rules) that can be understood by humans and enforced by machines. The choice of language for implementation is in principle, arbitrary. We initially used the Schematron [35,36] approach but have since moved to XSLT making heavy use of XPath, an extremely expressive language. XSLT has the advantage that it is implemented in all major languages and highly portable.

CMLLite has to support documents in a rigorous manner whilst accepting that these could come from a variety of sources and describe a wide range of possible chemical concepts. Therefore any CML element in the schema should be allowed, but would not by default have specific constraints. Any foreign XML elements would also be allowed and again would not have any specific constraints.

• An element can have text-only or element-only content (which may be empty, but there are no specifically empty models). For elements described in a defined convention constraints may apply. There are no restrictions on the order of elements in most content models.

• A document MAY contain more than one convention. Conventions are allowed to mandate other conventions.

• An element not specifically mentioned in a convention is effectively ignored by any tools that process after validation has succeeded (*i.e*. treated as any other foreign element), but is not removed from the document.

• Attribute data types are validated by their constraints in the CML schema but further constraints including *e.g*. required/forbidden, scoping of uniqueness and co-occurrence MAY be specified by conventions. These may restrict, but not alter the schema3 interpretation.

The interpretation of an element should not normally be affected by a convention. It constrains inputs and outputs but not the meaning of concepts. For example the atom/@x3 attribute always defines Cartesian coordinates, and in a right-handed system. A convention can insist that they do or do not exist, that other nodes must or must not exist, but it cannot change the primary semantics.

## Methodology of Validation

### Validation-driven Development

Our approach to validation is strongly informed by test-driven development (TDD), a well-used methodology for building modern software systems [37]. The schema and the validator have been built by creating tests and refining the schema and software such that the tests produce conformant behaviour. To illustrate the philosophy of TDD, we show a typical unit test before describing the construction of the validator. There are thousands of unit tests using CML in JUMBO, JUMBO-Converters [38], Bioclipse [39], CDK, Open Babel, *etc*.).

### A typical example of TDD

The following XML and Java snippets define the semantics of moleculeTool.getAverageBondLength() using the JUnit [40] framework:

<molecule id = 'mol5'>

<atomArray>

<atom id = 'a1' elementType = 'C' x3 = '0.0' y3 = '0.0' z3 = '0.0'/>

<atom id = 'a2' elementType = 'N' x3 = '0.0' y3 = '1.3' z3 = '0.0'/>

<atom id = 'a3' elementType = 'O' x3 = '1.0' y3 = '2.2' z3 = '0.0'/>

<atom id = 'a4' elementType = 'H' x3 = '0.85' y3 = '-0.54' z3 = '0.5'/>

<atom id = 'a5' elementType = 'H' x3 = '-0.85' y3 = '-0.54' z3 = '0.5'/>

</atomArray>

</molecule>

The function is described in words (in this case the method name is sufficient) and we implement a test which runs the code against an expected valid output:

@Test

**public void **testGetAverageBondLength() {

molTool5.calculateBondedAtoms();

Assert.*assertEquals*("average length", 1.2235,

molTool5.getAverageBondLength(CoordinateType.*CARTESIAN*),.0001);

}

This test passes the assertEquals statement if it can calculate the averageBondLength and also if the result is equal to the expected values within a given tolerance (0.0001). The test gives an example of conformant input and besides being a useful pedagogic and reference document it also implicitly defines semantics ("an average bond length calculation requires all atoms to have 3-D coordinates; these can be supplied as x3/y3/z3").

### General aspects of TDD

Test-driven development not only provide a method for verifying the behaviour of existing software-it also provides examples of typical use cases for anyone using CML. Note that unit tests provide implicit rather than explicit semantics-we can define any number of valid input and the outputs required for these, but the actual transformation can be performed by any means.

### Schemas and CMLValidator

Schemas and conventions are systems to validate documents (*validators*). The basic strategy used throughout the validator design process is to create documents to test them (*validatorTests*). The choice of tests is critical-ideally the implementer should think of every possible distinct case, but in practice this is reduced to generic cases. It is important to generate broken documents as well as valid ones, and this is often surprisingly difficult. In practice edge cases crop up unexpectedly in large corpora and these must then be added to the validatorTests. Appendix B shows the effort required to create tests for even a simple convention.

Once the validatorTests are created the convention or schema is then coded. In line with test-driven development this starts with the tests failing (deliberately) as there is no code. The validator is then coded until it passes the tests. Frequently during this process the author will gain insight and inspiration and refine the validatorTests.

### A schema3 validatorTest

**Figure 3:**
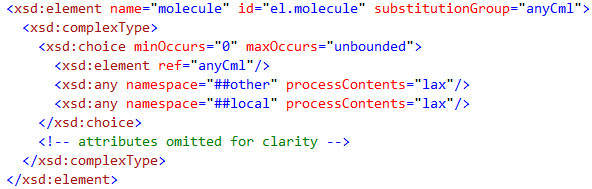
**A snippet from schema3 showing the typical relaxed content model of the molecule element container**.

As an example of how to test schema3 we take the definition of molecule in schema3 (Figure 3).

This is read as:

*A molecule can have zero (minOccurs = 0) or many (maxOccurs = unbounded) child elements in any order; it has no mixed content. The children can be any CML elements ("anyCml"), any elements with a foreign namespace (#other or #local (default namespace when not CML)). All the elements in the CML namespace are part of the anyCml substitution group*.

There are currently *ca*. 300 validatorTests (which test both schema and conventions) and we show examples that can validate the schema snippet for molecule. Each validatorTest is run against the schema, which only emits messages for invalid constructs.

(i)

<cml:molecule xmlns:cml = "http://www.xml-cml.org/schema">

<element-in-default-namespace>

This is fine. The null prefix is not bound

to anything and therefore is associated with

the default namespace

</element-in-default-namespace>

</cml:molecule>

This is valid because the null prefix is not explicitly bound to anything and therefore associated with the default namespace and the CML namespace is bound to the cml prefix. This construct is permitted because of the xsd:any namespace = '##local' in the schema.

(ii)

<molecule xmlns = "http://www.xml-cml.org/schema "xmlns:other = "http://www.example.net">

<other:foreign-element>

This is fine. The null prefix is bound to the

CML namespace and the "other" prefix

is bound to a non-CML namespace

</other:foreign-element>

</molecule>

This document is valid because the null prefix is bound to the CML namespace and the other prefix is bound to a non-CML namespace. This construct is permitted because of the xsd:any namespace = '##other' in the schema.

(iii)

<molecule xmlns = "http://www.xml-cml.org/schema">

<non-cml-element>

This is invalid. The null prefix is bound

to the CML namespace and the element

"non-cml-element" is not part of this

</non-cml-element>

</molecule>

This document is *not *valid because the null prefix is bound to the CML namespace and the element non-cml-element does not appear in the schema which defines CML.

(iv)

<cml:molecule xmlns:cml = "http://www.xml-cml.org/schema">

<cml:non-cml-element>

This is invalid. The cml prefix is bound

to the CML namespace and the element

"non-cml-element" does not form part of this

</cml:non-cml-element>

</cml:molecule>

This document is *not *valid because the cml prefix is bound to the CML namespace and the element non-cml-element does not appear in the schema which defines CML.

### CMLValidator report language

Because we have taken a unit-test-based approach the initial design of our convention verification software used Schematron, an ISO Standard for testing assertions about the structure of XML documents. After initial testing we found that Schematron scaled poorly with the complexity of the rules and was difficult to debug. We also desired a report language that could better support partial validation required to reflect the MUST, SHOULD, MAY approach to defining rules adopted by CMLLite.

**Figure 4:**
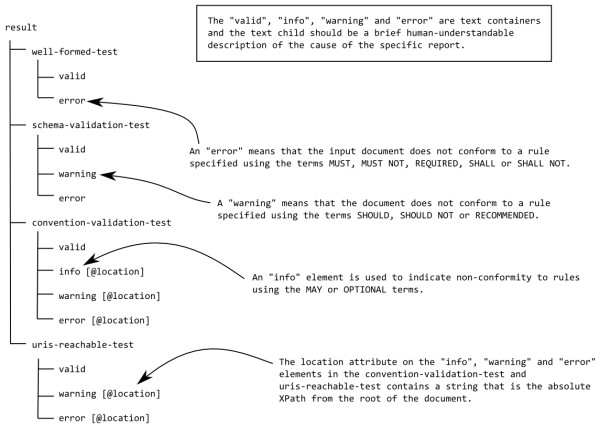
**An outline of the CML report language**. If a test (*e.g*. well-formed-test or URIs-reachable-test) contains a valid element child then it MUST NOT contain any warning or error element children. There is no such restriction on info elements and these may occur for input documents that otherwise conform completely to the convention.

The validating rules are now expressed directly as XSLT which gives greater flexibility and control structure. To support the MUST, SHOULD, MAY style of rules we have developed a small report language (Figure 4) to indicate the different levels of severity.

**Figure 5:**
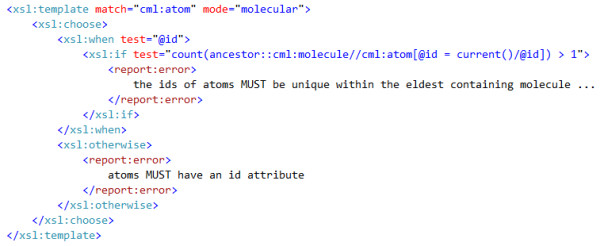
**Example rules expressed in XSLT: an atom must have an id attribute and the value of the id must be unique among the ids of all the atoms in the eldest containing parent molecule**. The cml prefix is bound to http://www.xml-cml.org/schema and the report prefix is bound to http://www.xml-cml.org/report/. The error reporting has been simplified for clarity (the location attribute is omitted).

Figure 5 shows how we use XSLT to encode a typical rule in the molecular convention:

• An atom must have an id attribute.

• The value of the id of an atom must be unique within the eldest containing molecule.

The XPath expression

count(ancestor::cml:molecule//cml:atom[@id = current()/@id] > 1)

can be decomposed into a set of steps which define a set of elements to query over and the query itself:

• ancestor::cml:molecule selects any molecule element of which the current atom is a descendent (child, grandchild *etc*.).

•//cml:atom then selects every single atom element that is a descendent of any of the set of molecules. Note that this must by definition include the original atom.

• [@id = current()/@id] restricts the set of atoms to only include those that have an id that is identical to the original atom (matched in the template).

• The count(...) > 1 expression forms the query and evaluates the number of atoms left in the set. If this is greater than 1 then multiple atoms in the same ancestor molecule have the same id.

The conventions in CMLLite have built-in rules which are generally not explicitly stated in the specification of conventions:

• A convention is applied through an element carrying the convention attribute. The convention applies to that element and all its descendants.

• The value of the convention attribute MUST be a QName that expands to the URI identifying the convention to be applied.

• A convention can require other conventions which must be explicitly specified on appropriate elements.

• If no conventions are declared a warning is issued.

We do not intend conventions to replace the CML schema and they are not a general schema language.

CMLValidator uses normal XSLT processing rules but makes special use of the mode attribute to allow validation of different conventions within the same document. < apply-templates mode = "*mode-name*" > limits subsequent validation to templates with mode = "mode-name". An apply-templates call without a mode will only call those templates without a mode (*i.e*. not governed by a convention in the document).

### An example-simpleUnit

The current conventions contain many hundreds of validatorTest and to illustrate them we create a very simple sub-convention: simpleUnit. There is already a mature convention for units using the schema3 elements unitList and unit. (Schema3 also defines a variety of attributes on unit which are still relevant but as they have default schema3 semantics they do not need explicit redefinition.) simpleUnit explores a small portion of this.

### The ruleset

1 The simpleUnit convention is specified with the http://www.xml-cml.org/convention/simpleUnit namespace.

2 The simpleUnit convention MUST be specified on a cml:unitList element using the convention attribute.

3 A cml:unitList element MUST contain at least one cml:unit child element.

4 A cml:unitList element MAY contain other child elements from the CML namespace or from foreign namespaces.

There are no constraints on where in a document the unitList element may appear.

### ValidatorTest

We start by creating an exhaustive set of tests against which the validator will be developed. These tests (Appendix B) are independent of the actual implementation of the validator. We can be confident that any validator that passes all these tests is likely to be useful in determining whether any of a wide range of documents is valid or invalid against the simpleUnit convention.

### Validator

**Figure 6:**
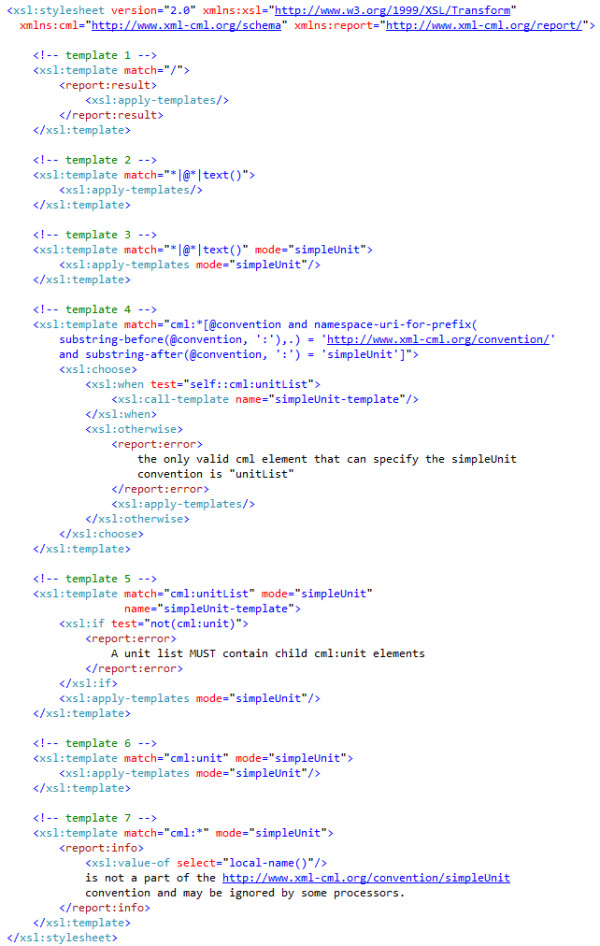
**the XSLT for the simpleUnit convention-the error reporting has been simplified for clarity**.

Figure 6 shows the XSLT required to encode the ruleset of the simpleUnit convention.

We now address the purpose of each of the templates in the validator in detail.

**Template 1**: creates the root report:result element

**Template 2**: match = "*|@*|text()" matches any element, attribute or text node when not in simpleUnit mode. The match expressions for the three node types are the most general possible and will therefore be overridden by any more specific matches. This template takes no action but allows recursive traversal to find elements covered by the simpleUnit conventions arbitrarily deep in the input document.

**Template 3**: carries out the same operations as template 2 but only when in simpleUnit mode. Non-CML elements may be interspersed with CML in the text document and will not cause the validator to emit warnings.

**Template 4**: Only elements from the cml namespace will be matched; the element MUST have a convention attribute, with namespace http://www.xml-cml.org/convention/ and the local name simpleUnit. The schema enforces that the value of the convention attribute must be a namespaceRefType.

If the element matched is cml:unitList this triggers mode = "simpleUnit" which remains in scope for all descendants.

If the element matched is not cml:unitList the validator informs the users that it is an error to specify the simpleUnit convention and apply-templates is called but not in simpleUnit mode.

**Template 5**: matches any cml:unitList element. If this does not have at least one cml:unit child element then an error is reported. Any child nodes are then processed in simpleUnit mode.

**Template 6**: matches any cml:unit element in simpleUnit mode. XSLT rules dictate that it has higher priority than template 7.

**Template 7**: matches any element from the CML namespace in simpleUnit mode. The match is more specific than template 3 but less specific than templates 4, 5 and 6. This will therefore catch any CML namespaced elements other than unitList and unit. The elements matched by this template are covered by rule 4-they are allowed but they are not really part of the convention, hence the output contains information to this effect.

This template is primarily for information, not errors-it is therefore appropriate to warn when CML elements might be ignored. Note that the presence of report:info elements in the report document does not mean that the input document is invalid.

## Interaction and extension of conventions

**Figure 7:**
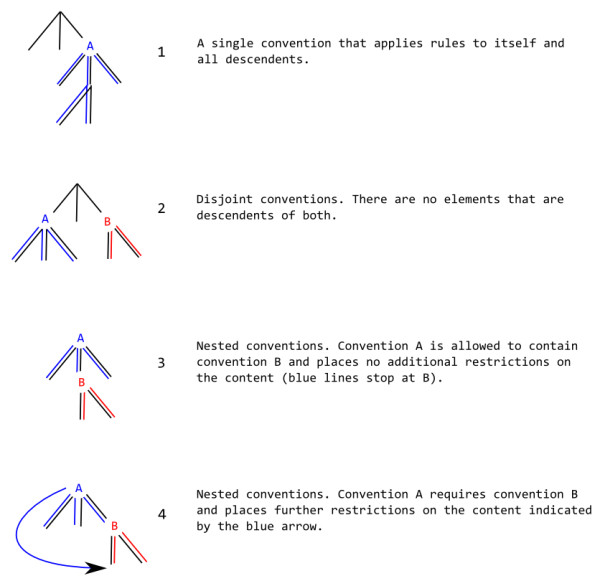
**Documents with multiple conventions**. The black lines represents the XML tree (DOM) and are shadowed by constraints imposed by conventions A (blue) and B (red).

Conventions are generally designed so that they can be mixed in a document, typically as discrete sections of a document (*i.e*. they do not overlap (instance 2 in Figure 7)). Thus the CMLSpect convention does not involve molecular, and molecular does not involve CMLSpect. The CMLValidator will engage the appropriate modes when processing each convention.

It is sometimes desirable to nest conventions (a subtree with one convention being found completely within a larger tree with a different convention-*e.g*. instances 3 and 4 in Figure 7). We use this approach in the current CMLLite conventions (Table 1) which may (recursively) validate subtrees labelled as having known conventions. The rules for nesting are under community review and Figure 7 shows the currently allowed interaction of conventions. The scope of a convention is thus similar to that of a namespace in that it "extends from the beginning of the start-tag in which it appears to the end of the corresponding end-tag" [41].

Some of the specifications from the molecular convention http://www.xml-cml.org/convention/molecular are given below;

• A molecule MUST contain at least one of the following elements: molecule, atomArray, name, label, formula.

• A molecule MUST NOT contain both a child molecule and a child atomArray.

• An atomArray MUST contain at least one atom.

• A molecule MAY contain zero or one bondArray children and a bondArray MUST contain at least one bond.

• Every atom MUST have an id which is unique within the eldest containing parent molecule.

• If an atom has an x3 coordinate it MUST also have y3 and z3 (and similarly if y3 or z3 are present).

The compchem convention http://www.xml-cml.org/convention/compchem has been developed as part of the Quixote project. It requires that the *initialization module *contains exactly one molecule and that all the atoms in this molecule MUST have three dimensional coordinates. Rather than create a new convention for molecules it was decided that these requirements were compatible with the molecular convention but required a tightening of some constraints.

Some of the rules from the compchem convention are shown below:

• There MUST be an initialization module which is a module element with a specific value of its dictRef attribute (cml:module/@dictRef = 'compchem:initialization' where the compchem prefix is bound to http://xml-cml.org/dictionary/compchem/).

• The initialization module MUST contain exactly one molecule.

• molecules MUST declare that they conform to the molecular convention by declaring this in the convention attribute (cml:molecule/@convention = 'conventions:molecular' where the conventions prefix is bound to http://www.xml-cml.org/convention/).

• The molecule in the initialization module is REQUIRED to have an atomArray child.

• All the atoms in the molecule in the initialization module MUST have three dimensional coordinates.

**Figure 8:**
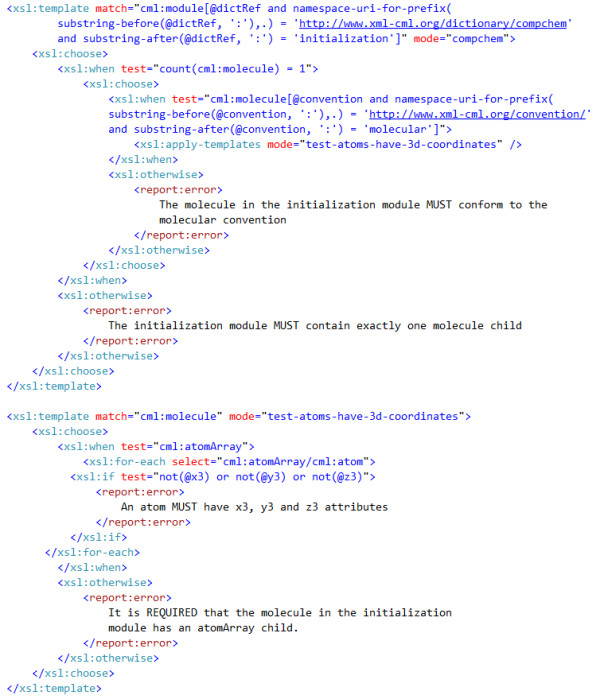
**A snippet showing how the compchem convention can rely on the molecular convention and add further restrictions**.

Figure 8 shows the part of the XSLT that will enforce the requirements on the molecule in the initialization module described above. The first template tests that there is only a single molecule child of the initialization module and that the molecule must specify the molecular convention. The existence of this convention statement will trigger the CMLValidator to apply the relevant rules to the molecule and its descendant nodes.

The second template is in a separate mode (test-atoms-have-3d-coordinates) and tests that an atomArray is present and that all the child atoms of this have 3D coordinates. Note that we do not need to check whether or not the molecule has child molecules or other atomArray children because this will be done by the molecular convention.

### Validation Service

Following the W3C validation tools [42] (specifically Unicorn [43]), we have created the CML validation service [44]. The validator is available in the following forms: an interactive form-based [45] webpage, a RESTful [46] web service and as a Java library.

The Java library is the same as the backend engine for the web-based services. The program consists of validator classes, an overall workflow control class and a ValidationReport class. The validation report class encapsulates both an XML document containing information about which tests have passed, failed or caused warnings and a ValidationResult property. The ValidationResult can be VALID, VALID_WITH_WARNINGS or INVALID.

The checks performed by the validator are shown below in order of application. If a particular check results in an INVALID ValidationResult no further processing is performed and the ValidationReport is created and returned.

1. It is well-formed XML. The control class can takes as input either an *InputStream *or a *nu.xom.Document *(xomDoc) and produces a ValidationReport. If input is an InputStream the program checks that it is well-formed XML (this is not necessary for a xomDoc as it is necessarily well-formed). A xomDoc is built from the InputStream and further processing is identical regardless of input format.

2. The xomDoc conforms to the CML schema3.

3. Deprecated constructs are not used. The use of deprecated constructs will give a VALID_WITH_WARNINGS result.

4. Any conventions specified in the document are obeyed.

5. All the prefixes used in namespaceRefTypes (effectively QNames) have been bound to namespaces and are resolvable URLs.

**Figure 9:**
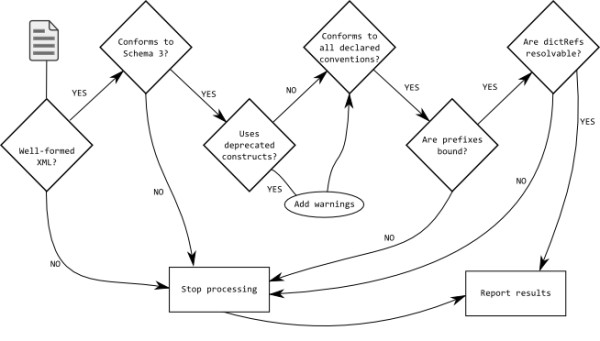
**Workflow of validation in the CMLValidator**.

**Figure 10:**
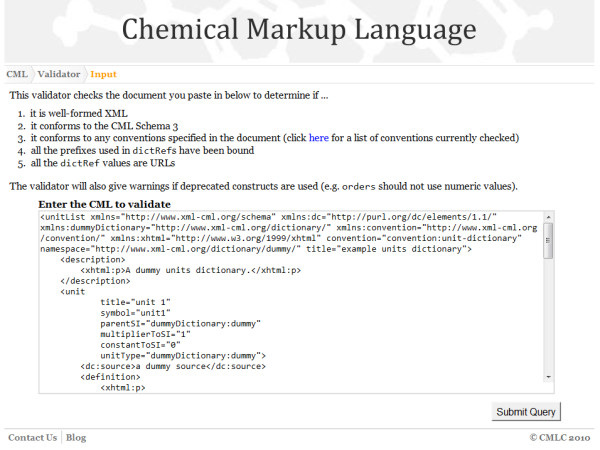
**The CMLValidator webform interface**. The input claims that it should conform to the unit-dictionary convention but unit 1 does not have an id attribute.

The final check has been put in place as a reminder to users that sharing information is preferable and they can only "code to the green bar" [37] by making their dictionaries and conventions *etc*. publicly available. The workflow is shown in Figure 9 and 10.

The RESTful webservice implementation is accessed by POSTing the XML/CML document to http://validator.xml-cml.org/validate which returns a ValidationReport. This must then be queried by the user to determine whether the overall validation resulted in VALID, VALID_WITH_WARNINGS or INVALID. Informal feedback from users indicated that it was more useful to send the complete ValidationReport rather than just a ValidationResult as feedback as this would allow the calling tool to do more.

**Figure 11:**
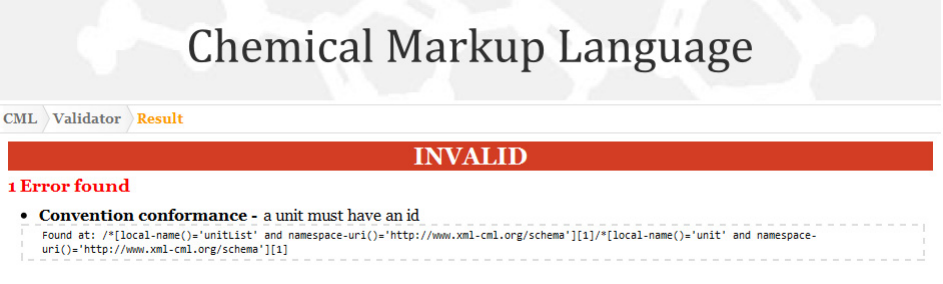
**Part of the CMLValidator results page showing that the input (in Figure 10) was invalid**. The report contains a human-understandable message and an XPath (machine-understandable) expression giving the location of the error.

**Figure 12:**
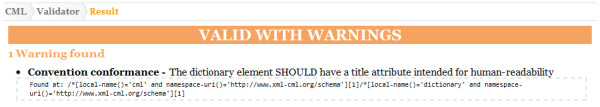
**Part of the CMLValidator results page showing that the input is valid but has warnings *i.e***. the convention states that the dictionary element SHOULD have a title but none was found in the document. The warning gives a human-understandable message and an XPath (machine-understandable) expression giving the location of the warning.

**Figure 13:**
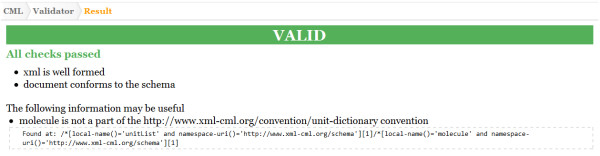
**Part of the CMLValidator results page showing that the document is valid and which checks have been performed**. Further information is also given because the input document contained a molecule element (which is not part of the unit-dictionary convention).

The website is effectively an instance of a tool that uses the RESTful implementation to do the actual validation but then interprets the results and displays them in the most human-user friendly fashion. Figures 11, 12 and 13 show the interactive form-based service in use.

## Conclusions

We have developed an approach to extensible semantics for Chemical Markup Language, where we assume that the current schema (schema3) is stable and expressive. There is enough software and data that this approach has been widely deployed and tested, even if it is not yet mainstream. Semantics are defined in the XSD schema, with additional natural language and validated using a unit test approach (Java and .NET). It works in the main fields of chemistry for which CML has been developed (molecules, reactions/syntheses, crystallography/solid-state, spectroscopy and computational chemistry). The approach encourages sub-communities in chemistry to create *conventions *which can be as rigid or fluid as they wish. The conventions can be rigorously unit tested using CMLValidator.

The convention-based approach is intermediate between natural language and formal systems. It relies, in part, on the wider community agreeing the semantics in schema3 (in several years deployment we have not yet had any disagreement with the basic elements and attributes and unit-tested examples). Sub-communities are starting to build their extensions of which the compchem convention being developed by the Quixote project is a prime example.

We believe the convention-based approach will help developers to create better software quicker. The tests/conventions define clear, testable APIs and these are essential for any distributed development.

The system interoperates fully with RDF-based systems. Many elements (especially value containers) can be algorithmically translated to RDF. A few core elements (primarily molecule, spectra, crystal) can be held in a more atomic form with bespoke semantics and software (it is, however, always possible to map into the details of these using URIs and to provide fine-grained links). By using this mixture of approaches we believe this is a cost-effective approach to interoperability within chemistry for those who wish to interoperate.

## Availability of Code

The CMLValidator and associated tests are available at http://bitbucket.org/cml/cmllite-validator-code and the web-based implementation is available at http://bitbucket.org/cml/cmllite-validator-ws both under an Apache 2.0 licence.

## Competing interests

The authors declare that they have no competing interests.

## Authors' contributions

JAT developed the CMLLite approach, created the CMLValidator and the test corpus, and wrote the manuscript. PMR is the original author of CML, created the test corpus and wrote the manuscript.

## Appendix A

The attributes in CML are defined in attributeGroups which must have unique names allowing them to be disambiguated within the schema. The attributeGroup defines an attribute, its datatype/allowed values, and the name of the attribute in the document (these do not have to be unique).

Element declarations in the schema specify which attributeGroups are allowed on them (which in this case caused polymorphism).

Table 2 shows all the attributes in CML schema3 that appear in the document as type. Values in "quotes" are enumerated allowed values, xsd:string means that any string content is permitted.

**Table 2:** All the attributes in CML schema3 that appear in the document as type.

Attribute Group name	On element	Allowed values
cellParameterType	cellParameter	"length" "angle"

peakStructureType	peakStructure	"coupling" "splitting" "other"

reactionStepList	reactionStepList	"unknown" "consecutive" "choice" "simultaneous" "other"

reactionType	reaction, reactionScheme	"chainReaction" "initiation" "termination" "reversible" "other"

spectrumType	spectrum	"infrared" "massSpectrum" "NMR" "UV/VIS" "other"

substanceListType	substanceList	"solution" "mixture" "other"

Type	action, actionList, eigen, list, object, observation, particle, substance	xsd:string

## Appendix B

The documents below are a subset of the documents used to test the behaviour of the simpleUnit convention validator. After every alteration (new test, bug fix *etc*.) of the convention, the validator is run against this test set to verify that its behaviour still conforms to expectations.

In all the examples below the cml prefix is bound to http://www.xml-cml.org/schema and the conventions prefix is bound to http://www.xml-cml.org/convention/.

### Documents Valid Against the simpleUnit Convention

The input documents below should all result in a ValidationResult of VALID and the ValidationReport MUST NOT contain info elements. The tests in this section are primarily concerned with ensuring that the convention is recognised wherever it appears in a document and that non-CML elements do not give rise to info reports.

(i)

<cml:unitList convention = "conventions:simpleUnit">

<cml:unit/>

</cml:unitList>

This produces the following ValidationReport:

<report xmlns = "http://www.xml-cml.org/report/">

<well-formed-test>

<valid >xml is well formed</valid>

</well-formed-test>

<schema-validation-test>

<valid>document conforms to the schema</valid>

</schema-validation-test>

<convention-validation-test>

<valid>document conforms to all the conventions specified</valid>

</convention-validation-test>

<uris-reachable-test>

<valid>All appropriate URIs were reachable</valid>

<valid>all dictRefs are resolvable </valid>

</uris-reachable-test>

</report>

The subsequent inputs produce exactly the same report and we therefore choose to explain what the document is testing for rather than show the output.

(ii)

<x:p xmlns:x = "http://www.w3.org/1999/xhtml">

<cml:unitList convention = "conventions:simpleUnit">

<cml:unit/>

</cml:unitList>

</x:p>

Tests that simpleUnit convention can be declared on a unitList that is not the root element of the document and is a child of a foreign namespaced element.

(iii)

<cml:module>

<cml:unitList convention = "conventions:simpleUnit">

<cml:unit/>

</cml:unitList>

</cml:module>

The simpleUnit convention can be declared on a unitList that is not the root element of the document and is a child of a CML element (module).

(iv)

<element-in-default-namespace>

<cml:unitList convention = "conventions:simpleUnit">

<cml:unit/>

</cml:unitList>

</element-in-default-namespace>

Tests that simpleUnit convention can be declared on a unitList that is not the root element of the document and is a child of an element from the default-namespace.

(v)

<x:p xmlns:x = "http://www.w3.org/1999/xhtml">

the unitList need not be the root element

<cml:unitList convention = "conventions:simpleUnit">

<cml:unit/>

</cml:unitList>

</x:p>

Test that although CML does not have a mixed content model the unitList can occur within non-CML mixed content.

(vi)

<x:p xmlns:x = "http://www.w3.org/1999/xhtml">

there are multiple instances of the simpleUnit

convention in this document

<cml:unitList convention = "conventions:simpleUnit">

<cml:unit/>

</cml:unitList>

<cml:unitList convention = "conventions:simpleUnit">

<cml:unit/>

</cml:unitList>

</x:p>

Test that there can be multiple disjoint simpleUnit convention declarations in the same document.

(vii)

<cml:unitList convention = "conventions:simpleUnit">

<cml:unit/>

<x:p xmlns:x = "http://www.w3.org/1999/xhtml">

non cml child-this is fine

</x:p>

</cml:unitList>

Tests that the unitList element can contain foreign namespaced elements without giving rise to info reports.

(viii)

<cml:unitList convention = "conventions:simpleUnit">

<element-in-default-namespace/>

<cml:unit/>

</cml:unitList>

Tests that the unitList element can contain elements from the default namespace without giving rise to info reports.

(ix)

<cml:unitList convention = "conventions:simpleUnit">

<cml:unit>

<x:p xmlns:x = "http://www.w3.org/1999/xhtml">

non cml child-this is fine

</x:p>

</cml:unit>

</cml:unitList>

Tests that the unit element can contain foreign namespaced elements without giving rise to info reports.

(x)

<cml:unitList convention = "conventions:simpleUnit">

<cml:unit>

<element-in-default-namespace/>

</cml:unit>

</cml:unitList>

Tests that the unit element can contain elements from the default namespace without giving rise to info reports.

(xi)

<cml:unitList convention = "conventions:simpleUnit">

<cml:unit/>

<cml:unit>

<x:p xmlns:x = "http://www.w3.org/1999/xhtml">

multiple cml:unit elements are allowed

</x:p>

</cml:unit>

</cml:unitList>

Tests that a unitList may contain more than one unit child.

### Documents Valid (with info reports) Against the simpleUnit Convention

The input documents below should all result in a ValidationResult of VALID and the ValidationReport should contain a single info element and MUST NOT contain either error or warning elements. info elements are used to give information about rules in a convention involving the MAY clause. Note that the complete ValidationReport is given for the first example but subsequent examples only contain the error message for brevity.

(i)

<cml:unitList convention = "conventions:simpleUnit">

<cml:unit/>

<cml:molecule/>

</cml:unitList>

Produces:

<report xmlns = "http://www.xml-cml.org/report/">

<well-formed-test>

<valid > xml is well formed </valid>

</well-formed-test>

<schema-validation-test>

<valid > document conforms to the schema </valid>

</schema-validation-test>

<convention-validation-test>

<info location = "/*[local-name() = 'unitList' and namespace-uri() = 'http://www.xml-cml.org/schema'] [1]/*[local-name() = 'molecule' and namespace-uri() = 'http://www.xml-cml.org/schema'] [1]">

molecule is not a part of the http://www.xml-cml.org/convention/simpleUnit convention and may be ignored by some processors.

</info>

<valid>

document conforms to all the conventions specified

</valid>

</convention-validation-test>

<uris-reachable-test>

<valid > All appropriate URIs were reachable </valid>

<valid > all dictRefs are resolvable </valid>

</uris-reachable-test>

</report>

(ii)

<cml:unitList convention = "conventions:simpleUnit">

<cml:unit>

<cml:atom/>

</cml:unit>

</cml:unitList>

Produces: "atom is not a part of the http://www.xml-cml.org/convention/simpleUnit convention and may be ignored by some processors"

(iii)

<cml:unitList convention = "conventions:simpleUnit">

<cml:unit/>

<cml:unit>

<cml:bond/>

</cml:unit>

</cml:unitList>

Produces: "bond is not a part of the http://www.xml-cml.org/convention/simpleUnit convention and may be ignored by some processors"

Note that if the bond specified a numeric bond order (*e.g*. order = '1') the test result would be VALID_WITH_WARNINGS because numeric bond orders are deprecated.

(iv)

<cml:unitList convention = "conventions:simpleUnit">

<cml:unit>

<x:p xmlns:x = "http://www.w3.org/1999/xhtml">

this is still going to be processed in

unitList mode.

<cml:bond/>

</x:p>

</cml:unit>

</cml:unitList>

Produces: "bond is not a part of the http://www.xml-cml.org/convention/simpleUnit convention and may be ignored by some processors"

(v)

<x:p xmlns:x = "http://www.w3.org/1999/xhtml">

the unitList need not be the root element

<cml:unitList convention = "conventions:simpleUnit">

<cml:molecule/>

<cml:unit/>

</cml:unitList>

</x:p>

Produces: "molecule is not a part of the http://www.xml-cml.org/convention/simpleUnit convention and may be ignored by some processors".

### Documents Invalid Against the simpleUnit Convention

The documents below should all result in a ValidationResult of INVALID. The ValidationReport should contain a single error element and should not contain either info or warning elements.

(i)

<cml:molecule convention = "conventions:simpleUnit">

<cml:unitList>

<cml:unit/>

</cml:unitList>

</cml:molecule>

Produces the following ValidationReport:

<report xmlns = "http://www.xml-cml.org/report/">

<well-formed-test>

<valid > xml is well formed </valid>

</well-formed-test>

<schema-validation-test>

<valid > document conforms to the schema </valid>

</schema-validation-test>

<convention-validation-test>

<error location = "/*[local-name() = 'molecule' and namespace-uri() = 'http://www.xml-cml.org/schema'] [1]@*[local-name() = 'convention' and namespace-uri() = '']">

the only valid cml element that can specify the simpleUnit convention is "unitList"

</error>

</convention-validation-test>

<uris-reachable-test>

<valid > All appropriate URIs were reachable </valid>

<valid > all dictRefs are resolvable </valid>

</uris-reachable-test>

</report>

(ii)

<x:p xmlns:x = "http://www.w3.org/1999/xhtml">

<cml:molecule convention = "conventions:simpleUnit">

<cml:unitList>

<cml:unit/>

</cml:unitList>

</cml:molecule>

</x:p>

Produces: "the only valid cml element that can specify the simpleUnit convention is 'unitList'". (Illustrating that the document is still being correctly traversed.)

(iii)

<cml:unitList convention = "conventions:simpleUnit"/>

Produces: "A unit list MUST contain child cml:unit elements".

(iv)

<cml:unitList convention = "conventions:simpleUnit">

<!-- not valid, a unitList must have at least one unit child -->

</cml:unitList>

Produces: "A unit list MUST contain child cml:unit elements".

(v)

<cml:unitList convention = "conventions:simpleUnit">

<x:p xmlns:x = "http://www.w3.org/1999/xhtml">

no unit child of unitList

</x:p>

</cml:unitList>

Produces: "A unit list MUST contain child cml:unit elements".

(vi)

<cml:unitList convention = "conventions:simpleUnit">

<x:p xmlns:x = "http://www.w3.org/1999/xhtml">

<cml:unit/>

This unit is not a direct child of unitList

and therefore should cause an error.

</x:p>

</cml:unitList>

Produces: "A unit list MUST contain child cml:unit elements".

(vii)

<cml:unitList convention = "conventions:simpleUnit">

<cml:unitList>

<cml:unit>

<x:p xmlns:x = "http://www.w3.org/1999/xhtml">

the outer unitList does not have at least

one unit child

</x:p>

</cml:unit>

</cml:unitList>

</cml:unitList>

Produces: "A unit list MUST contain child cml:unit elements".

## References

[B1] Bauerschmidt S Gasteiger J Overcoming the Limitations of a Connection Table Description: A Universal Representation of Chemical Species J Chem Inf Comput Sci 1997 37 705 714 10.1021/ci9704423

[B2] Hughes G Mills H De Roure D Frey JG Moreau L Schraefel MC Smith G Zaluska E The semantic smart laboratory: a system for supporting the chemical *e*Scientist Org Biomol Chem 2004 2 3284 3293 10.1039/b410075a 15534706

[B3] Chepelev LL Dumontier M Chemical Entity Semantic Specification: Knowledge representation for efficient semantic cheminformatics and facile data integration J Cheminf 2011 3 20 10.1186/1758-2946-3-20 PMC3121712 21595881

[B4] Murray-Rust P Rzepa HS CML: Evolution and Design J Cheminf 2011 3 44 10.1186/1758-2946-3-44 PMC3205047 21999549

[B5] Chemistry Add-in for Word http://chem4word.codeplex.com/Accessed 2011-06-28

[B6] Chemical Markup Language http://sourceforge.net/projects/cml/Accessed 2011-06-28

[B7] Sankar P Alain K Aghila G Model Tool to Describe Chemical Structures in XML Format Utilizing Structural Fragments and Chemical Ontology J Chem Inf Model 2010 50 755 770 10.1021/ci100052b 20429589

[B8] Sankar P Aghila G Design and Development of Chemical Ontologies for Reaction Representation J Chem Inf Model 2006 46 2355 2368 10.1021/ci050533x 17125179

[B9] Resource Description Framework, RDF http://www.w3.org/RDF/Accessed 2011-06-28

[B10] RDF Vocabulary Description Language 1.0: RDF Schema http://www.w3.org/TR/rdf-schema/Accessed 2011-06-28

[B11] Web Ontology Language (OWL) http://www.w3.org/2004/OWL/Accessed 2011-06-28

[B12] Belleau F Nolin M-A Tourigny N Rigault P Morissette J Bio2RDF: Towards a mashup to build bioinformatics knowledge systems J Biomed Inf 2008 41 5 706 716 10.1016/j.jbi.2008.03.004 18472304

[B13] Adams SE de Castro P Echenique P Estrada J Hanwell MD Murray-Rust P Sherwood P Thomas J Townsend J The Quixote project: Collaborative and Open Quantum Chemistry data management in the Internet age J Cheminf 2011 3 38 10.1186/1758-2946-3-S1-P38 PMC3206452 21999363

[B14] Quixote project on QC databases http://quixote.wikispot.org/Accessed 2011-06-28

[B15] Adams N Cannon E Murray-Rust P ChemAxiom-An Ontological Framework for Chemistry in Science Nature Precedings 2009

[B16] Jones R MacGillivray M Murray-Rust P Pitman J Sefton P O'Steen B Waites W Open Bibliography for Science, Technology, and Medicine J Cheminf 2011 3 47 10.1186/1758-2946-3-47 PMC3206455 21999661

[B17] The Chemistry Development Kit, CDK http://sourceforge.net/projects/cdk/Accessed 2011-06-28

[B18] Open Babel: The Open Source Chemistry Toolbox http://openbabel.org/Accessed 2011-06-28

[B19] Davis FD Perceived usefulness, perceived ease of use, and user acceptance of information technology MIS Quarterly 1989 13 319 340 10.2307/249008

[B20] Postel J Postel's Law http://en.wikipedia.org/wiki/Jon_Postel#Postel.27s_Law Accessed on 2011-06-2821982672

[B21] IUPAC International Chemical Identifier, InChI http://www.iupac.org/inchi/Accessed 2011-06-28

[B22] XHTML specification http://www.w3.org/TR/xhtml11/Accessed 2011-06-28

[B23] Mathematical Markup Language (MathML) specification http://www.w3.org/TR/MathML2/Accessed 2011-06-28

[B24] W3C Scalable Vector Graphics (SVG) Working Group http://www.w3.org/Graphics/SVG/Accessed 2011-06-28

[B25] Demir E Cary MP Paley S Fukuda K Lemer C Vastrik I Wu G D'Eustachio P Schaefer C Luciano J Schacherer F Martinez-Flores I Hu Z Jimenez-Jacinto V Joshi-Tope G Kandasamy K Lopez-Fuentes A Mi H Pichler E Rodchenko I Splendiani A Tkachev S Zucker J Gopinath G Rajashima H Ramakrishnan R Shah I Syed M Anwar N Babur O Blinov M Brauner E Corwin D Donaldson S Gibbons F Goldberg R Hornbeck R Luna A Murray-Rust P Neumann E Reubenacker O Samwald M van Iersel M Wimalaratne S Allen K Braun B Whirl-Carrillo M Cheung K-H Dahlquist K Finney A Gillespie M Glass E Gong L Haw R Honig M Hubaut O Kane D Krupa S Kutmon M Leonard J Marks D Merberg D Petri V Pico A Ravenscroft D Ren L Shah N Sunshine M Tang R Whaley R Letovsky S Buetow KH Rzhetsky A Schachter V Sobral BS Dogrusoz U McWeeney S Aladjem M Birney E Collado-Vides J Goto S Hucka M Le Novère N Maltsev N Pandey A Thomas P Wingender E Karp PD Sander C Bader GD The BioPAX community standard for pathway data sharing Nature Biotechnology 2010 28 935 942 10.1038/nbt.1666 20829833 PMC3001121

[B26] García A Murray-Rust P Wakelin J The use of XML and CML in computational chemistry and physics programs Proceedings of the UK e-Science All Hands Meeting 2004 Engineering and Physical Sciences Research Council 1111 1114

[B27] Kuhn S Helmus T Lancashire RJ Murray-Rust P Rzepa HS Steinbeck C Willighagen EL Chemical Markup, XML, and the World Wide Web. 7. CMLSpect, an XML Vocabulary for Spectral Data J Chem Inf Model 2007 47 2015 2034 10.1021/ci600531a 17887743

[B28] Lancashire RJ The JSpecView Project: an Open Source Java viewer and converter for JCAMP-DX, and XML spectral data files Chem Cent J 2007 1 31 10.1186/1752-153X-1-31 18067663 PMC2203984

[B29] Bradner S IETF RFC 2119: Key words for use in RFCs to Indicate Requirement Levels http://www.ietf.org/rfc/rfc2119.txt Accessed 2011-06-28

[B30] Murray-Rust P Townsend J Adams SE Phadungsukanan W Thomas J The semantics of Chemical Markup Language (CML): dictionaries and conventions J Cheminf 2011 3 43 10.1186/1758-2946-3-S1-P43 PMC3206453 21999509

[B31] XML Schema Definition Language (XSD) 1.1 Part 1: Structures http://www.w3.org/TR/xmlschema11-1/Accessed 2011-06-28

[B32] XML Schema Definition Language (XSD) 1.1 Part 2: Datatypes http://www.w3.org/TR/xmlschema11-2/Accessed 2011-06-28

[B33] XSL Transformations (XSLT) http://www.w3.org/TR/xslt20/Accessed 2011-06-28

[B34] XML Path Language http://www.w3.org/TR/xpath/Accessed 2011-06-28

[B35] Schematron, a language for making assertions about patterns found in XML documents http://www.schematron.com/Accessed 2011-06-28

[B36] Tennison J XSLT UK 2001 Report http://www.xml.com/pub/a/2001/04/25/xsltuk.html?page=2 Accessed 2011-06-28

[B37] Kent B Test Driven Development: By Example 2002 Boston, Massachusetts: Addison-Wesley Professional

[B38] JUMBO-Converters https://bitbucket.org/wwmm/jumbo-converters Accessed 2011-06-28

[B39] Bioclipse http://www.bioclipse.net/Accessed 2011-06-28

[B40] Hunt A Thomas D Pragmatic Unit Testing in Java with JUnit 2003 Raleigh, NC: Pragmatic Bookshelf

[B41] Namespaces in XML http://www.w3.org/TR/xml-names/Accessed 2011-06-28

[B42] W3C Quality Assurance Tools http://www.w3.org/QA/Tools/Accessed 2011-06-28

[B43] Unicorn-W3C's Unified Validator http://validator.w3.org/unicorn/Accessed 2011-06-28

[B44] CMLValidator service http://validator.xml-cml.org/Accessed 2011-06-28

[B45] HTML4 Recommendation-Forms http://www.w3.org/TR/html4/interact/forms.html Accessed 2011-06-28

[B46] Fielding RT Architectural Styles and the Design of Network-based Software Architectures PhD thesis 2000 University of California, Irvine

[B47] Chempound repository http://quixote.ch.cam.ac.uk/Accessed 2011-06-28

